# Spreading of Pandemic *Vibrio parahaemolyticus* O3:K6 and Its Serovariants: A Re-analysis of Strains Isolated from Multiple Studies

**DOI:** 10.3389/fcimb.2017.00188

**Published:** 2017-05-18

**Authors:** Dongsheng Han, Fei Yu, Hui Tang, Chuanli Ren, Caiyun Wu, Pan Zhang, Chongxu Han

**Affiliations:** ^1^Clinical Medical Examination Center, Northern Jiangsu People's HospitalYangzhou, China; ^2^Key Laboratory of Clinical In Vitro Diagnostic Techniques of Zhejiang Province, Department of Clinical Laboratory, First Affiliated Hospital, College of Medicine, Zhejiang UniversityHangzhou, China; ^3^Department of Pathology and Pathophysiology, Institute for Brain Research, Collaborative Innovation Center for Brain Science, Huazhong University of Science and TechnologyWuhan, China

**Keywords:** *Vibrio parahaemolyticus*, multilocus sequence typing, pandemic clone, gastroenteritis, genetic diversity

## Abstract

In China, *V. parahaemolyticus* has been a leading cause of foodborne outbreaks and bacterial infectious diarrhea since the 1990s, and most infections have been associated with the pandemic *V. parahaemolyticus* O3:K6 and its serovariants. However, a comprehensive overview of the sero-prevalence and genetic diversity of the pandemic *V. parahaemolyticus* clone in China is lacking. To compensate for this deficiency, pandemic isolates in both clinical and environmental Chinese samples collected from multiple studies were analyzed in this study. Surprisingly, as many as 27 clinical pandemic serovariants were identified and were widely distributed across nine coastal provinces and two inland provinces (Beijing and Sichuan). O3:K6, O4:K68, and O1:KUT represented the predominant clinical serovars. Only four environmental pandemic serovariants had previously been reported, and they were spread throughout Shanghai (O1:KUT, O3:K6), Jiangsu (O3:K6, O4:K48), Zhejiang (O3:K6), and Guangdong (O4:K9). Notably, 24 pandemic serovariants were detected within a short time frame (from 2006 to 2012). The pandemic isolates were divided into 15 sequence types (STs), 10 of which fell within clonal complex (CC) 3. Only three STs (ST3, ST192, and ST305) were identified in environmental isolates. Substantial serotypic diversity was mainly observed among isolates within pandemic ST3, which comprised 21 combinations of O/K antigens. The pandemic O3:K6 serotype showed a high level of sequence diversity, which was shared by eight different STs (ST3, ST227, ST431, ST435, ST487, ST489, ST526, and ST672). Antimicrobial susceptibility testing revealed that most isolates shared similar antibiotic susceptibility profiles. They were resistant to ampicillin but sensitive to most other drugs that were tested. In conclusion, the high levels of serotypic and genetic diversity of the pandemic clone suggest that the involved regions are becoming important reservoirs for the emergence of novel pandemic strains. We underscore the need for routine monitoring to prevent pandemic *V. parahaemolyticus* infection, which includes monitoring antimicrobial responses to avoid excessive misuse of antibiotics. Further investigations are also needed to delineate the specific mechanisms underlying the possible seroconversion of pandemic isolates.

## Introduction

*Vibrio parahaemolyticus*, a Gram-negative bacterium, is a natural inhabitant of estuarine and coastal environments. In humans, this pathogen is a globally important cause of acute gastroenteritis. It's a multi-serotype pathogenic bacteria, and can be classified into 13 O serotypes and 71 K serotypes based on somatic (O) antigens and capsular (K) antigens (Han et al., 2008). Since 1997, the pandemic isolates, including serotype O3:K6 and its serovariants, have spread globally by either sporadic diarrhea or contaminated food-related outbreaks (Nair et al., 2007; Chowdhury et al., 2013).

All the pandemic isolates share the following specific genetic markers: the presence of the thermostable direct hemolysin(*tdh*) gene, the absence of the TDH-related hemolysin(*trh*) gene, and a distinctive *toxRS* sequence (*toxRS/new*), which can be amplified by a group-specific PCR (GS-PCR) (Matsumoto et al., 2000). Until 2007, 22 serotypes had been traced to the pandemic clone based on these characteristics (Nair et al., 2007). In recent years, an increasing number of pandemic serotypes have been reported to be widely distributed in countries across four continents (Asia, Europe, the Americas and Africa) (Ansaruzzaman et al., 2008; Ottaviani et al., 2010; Powell et al., 2013; Li et al., 2014; Velazquez-Roman et al., 2014; Guerrero et al., 2017), suggesting that these pandemic serotypes pose a mounting public health threat. This threat calls for higher surveillance of the pandemic clone to reduce illnesses.

In China, *V. parahaemolyticus* has been the leading cause of foodborne outbreaks and bacterial infectious diarrhea since the 1990s, especially in coastal regions (Gao et al., 2016; Li et al., 2016). The pandemic serotype O3:K6 was first documented as the dominant serotype in 2002 and was proven to be a pandemic clone in 2008 (Vongxay et al., 2008). During the period of 2007–2012, approximately 56% of the clinical isolates in southern coastal areas of China had pandemic characteristics (Li et al., 2014). In one of our multi-center active surveillance programs, we found that 63.3% of the tested isolates were pandemic isolates in southeastern China from 2009 to 2013 (Chen et al., 2016).

The above findings indicate that the pandemic clone of *V. parahaemolyticus* plays an important role in causing infectious diseases in China. However, although laboratory-based surveillance for acute infectious diarrhea has been established in several coastal regions (Yu et al., 2011; Zhang et al., 2014; Li et al., 2015), a nationwide study or review of the distribution of infections caused by the pandemic isolates of *V. parahaemolyticus* has not been conducted in this country. Thus, a full understanding of the spread of this unique clone is needed to prevent outbreaks and sporadic illnesses in China.

In this study, we identified Chinese pandemic isolates of *V. parahaemolyticus* mostly from published literatures, and re-analyzed the sero-prevalence and genetic diversity of these pandemic isolates as a whole. We isolated some of these pandemic isolates in our active studies of diarrheal infection, and mainly focused on their antimicrobial responses in this study, which has not been shown previously. Overall, our intention is to generate a comprehensive overview of the spread of pandemic *Vibrio parahaemolyticus* O3:K6 and its serovariants in China since the emergence of this clone.

## Materials and methods

### Datasets utilized in the present study

To identify as many available pandemic isolates of *V. parahaemolyticus* as possible, we conducted a comprehensive search of several databases, including the US National Library of Medicine, PubMed, Elsevier, Springer, and China National Knowledge Infrastructure. We searched for all relevant Chinese studies using combinations of the following terms (through October 1, 2016): “*Vibrio parahaemolyticus*,” “pandemic clone,” “pandemic strains,” “pandemic isolates,” “O3:K6 serotype,” and “O3:K6 clone.” Additional eligible studies were identified from references cited in the relevant articles. The full text of each potentially relevant paper was scrutinized, and a total of 290 representative clinical and environmental *V. parahaemolyticus* isolates (toxRS/*new*+, *tdh*+ and *trh*−) were extracted from 16 studies and selected as the research subjects of this investigation.

Among the 290 pandemic isolates, 120 are from our laboratory, including 98 ST3 isolates, 21 ST88 isolates, and one ST672 isolate. Most of the isolates include information about the sampling area, year of isolation, source, serotype and multilocus sequence typing. Details on the individual isolates are summarized in Additional file 1: Table S1.

**Figure 1:**
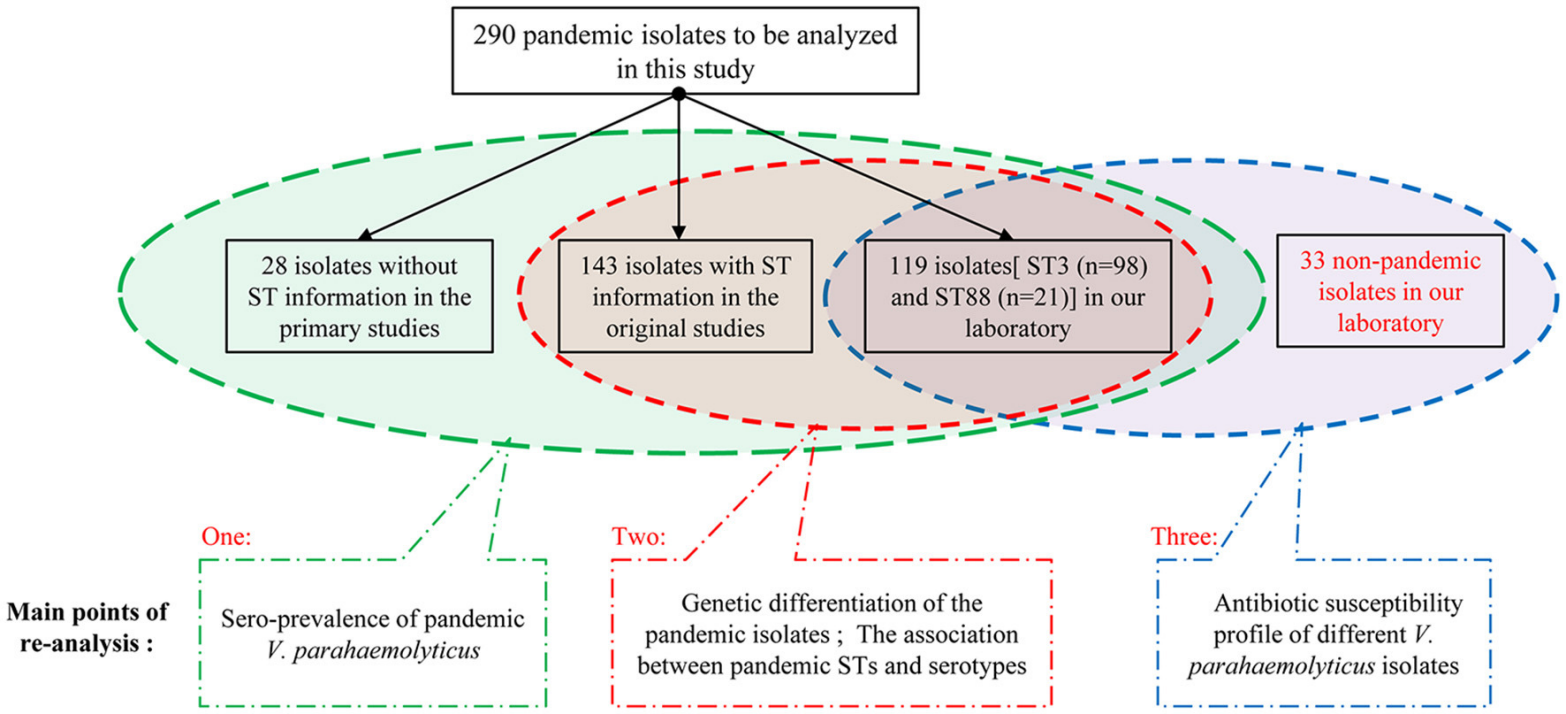
**The grouping of the collected isolates and the main points of re-analysis in this study**.

We grouped these 290 pandemic isolates according to the integrity of background information (Figure 1), and then carried out the re-analysis of the spreading of pandemic *V. parahaemolyticus* O3:K6 and its serovariants.

### Assignment to clonal complexes

Various typing methods have been used to distinguish *V. parahaemolyticus* isolates for epidemiological investigations (Marshall et al., 1999; Gonzalez-Escalona et al., 2008; Lüdeke et al., 2015). The high accuracy and repeatability of current sequencing technology, the ability to compare DNA sequences universally and the ability to share data among laboratories make multi-locus sequence typing (MLST) a complete, robust, and reliable typing method. In recent years, scientists have begun using whole genome sequencing (WGS) to analyze historical collections of isolates, providing new insights for understanding population dynamics among different *V. parahaemolyticus* isolates. However, many laboratories are not currently using WGS, as data are not easily shared among laboratories. For this reason, we used MLST to assign clonal complexes for the pandemic isolates collected in this study.

The MLST scheme used internal fragments of the seven house-keeping genes [*recA*_(729*bp*)_, *dnaE*_(557*bp*)_, *gyrB*_(592*bp*)_, *dtdS*_(458*bp*)_, *pntA*_(430*bp*)_, *pyrC*_(493*bp*)_, and *tnaA*_(423*bp*)_]. The standard amplification protocol was published on the *V. parahaemolyticus* MLST web site (http://pubmlst.org/vparahaemolyticus/). The allele designations and sequence types (STs) of all the selected isolates had been determined based on the variation of the seven genes. Based on the defined STs, all the pandemic isolates were compared using global optimal eBURST analysis (goeBURST) version 1.2.1 (http://www.phyloviz.net/goeburst/). Clonal complexes were conservatively defined as a cluster of STs, in which all STs were linked as single-locus variants (SLVs, two STs differing from each other at a single locus) to at least one other ST (Feil et al., 2004). The singleton STs corresponded to STs differing from the others by three or more of the seven loci (Esteves et al., 2015).

### Antimicrobial susceptibility testing

We selected the pandemic isolates [ST3 (*n* = 98) and ST88 (*n* = 21)] detected in our laboratory from the 290 isolates to be analyzed in this study and other 33 non-pandemic isolates identified in our laboratory to conduct antimicrobial susceptibility testing with 20 antimicrobial agents (Figure 1). The testing was performed using the disk diffusion method according to the Clinical and Laboratory Standards Institute (CLSI, 2010). *E. coli* ATCC25922 and *Staphylococcus aureus* ATCC25923 were employed as bacterial strains for quality control. Characterization of the resistance and susceptibility profile of the isolates was determined by measuring inhibitory zone, and then compared with the interpretative chart (Table 1).

**Table 1:** **The interpreted results of drug susceptibility testing**.

**Antimicrobial agent**		**Drug content (μg)**	**Bacteriostatic circle diameter (mm)**		
			**Susceptible (S)**	**Intermediate (I)**	**Resistant (R)**
Ampicillin	AMP	10	≥17	14–16	≤13
Amoxicillin-clavulanic acid	AMC	20/10	≥18	14–17	≤13
Ampicillin-salbactam	SAM	10/10	≥15	12–14	≤11
Piperacillin-tazobactam	TZP	100/10	≥21	18–20	≤17
Piperacillin	PIP	100	≥21	18–20	≤17
Cefazolin	CZO	30	≥18	15–17	≤14
Cefuroxime	CXM	30	≥18	15–17	≤14
Ceftazidime	CAZ	30	≥18	15–17	≤14
Cefotaxime	CTX	30	≥23	15–22	≤14
Cefepime	FEP	30	≥18	15–17	≤14
Cefotaxime	FOX	30	≥18	15–17	≤14
Imipenem	IPM	10	≥16	14–15	≤13
Meropenem	MEM	10	≥16	14–15	≤13
Amikacin	AMK	30	≥17	15–16	≤14
Gentamycin	GEN	10	≥15	13–14	≤12
Cefotaxime	CIP	5	≥21	16–20	≤15
Levofloxacin	LVX	5	≥17	14–16	≤13
Trimethoprim-Sulphamethoxazole	SXT	1.25/23.75	≥16	11–15	≤10
Tetracycline	TCY	30	≥19	15–18	≤14
Chloramphenicol	CHL	30	≥18	13–17	≤12

## Results

### Sero-prevalence of pandemic *V. parahaemolyticus*

**Figure 2:**
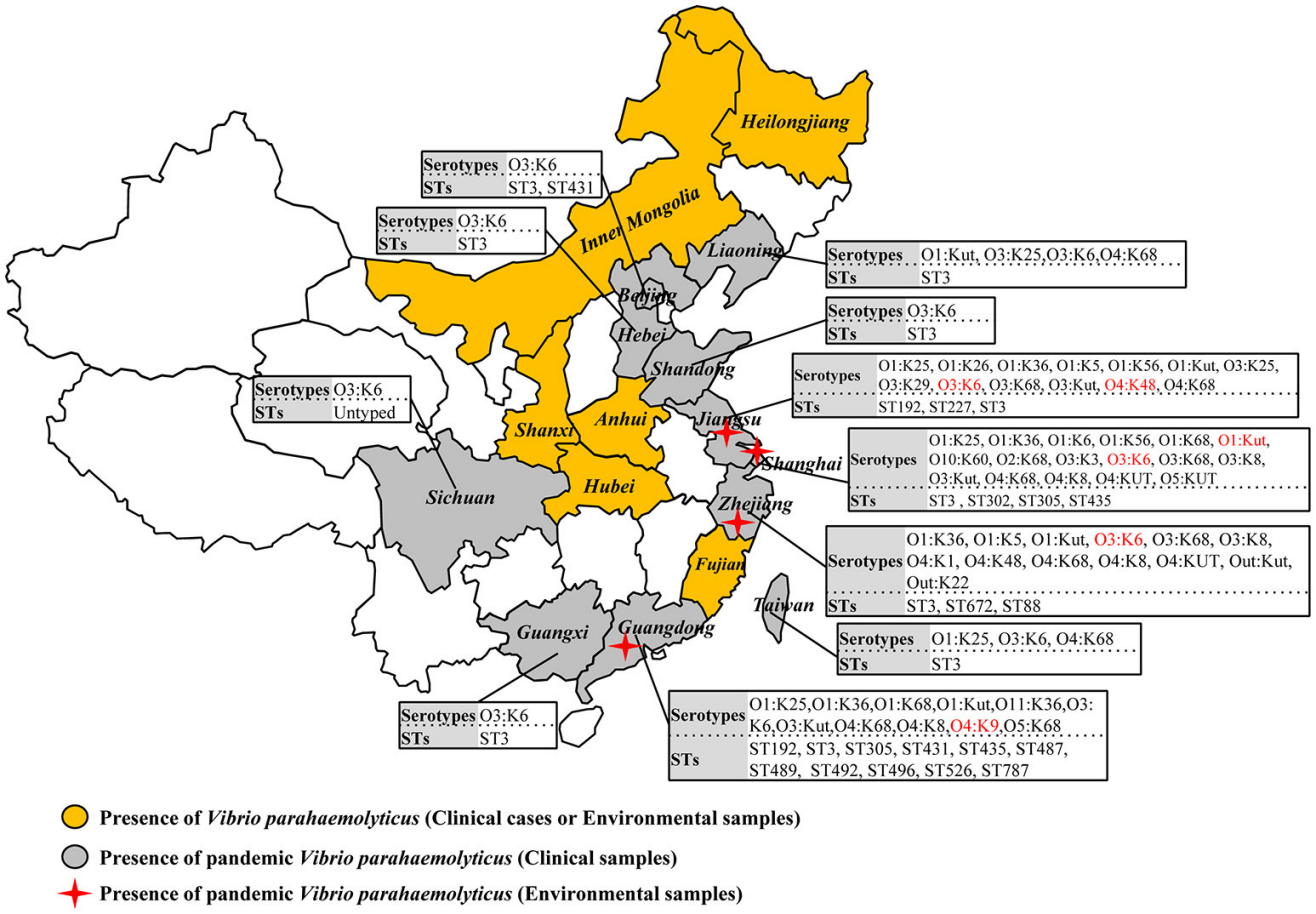
**Map showing the sero-prevalence and sequence diversity of clinical and environmental pandemic O3:K6 and its serovariants of *V. parahaemolyticus* found in Chinese samples**.

A comprehensive map of the dissemination of the pandemic serotypes detected in China was generated according to a detailed review (Figure 1). The pandemic serotypes were highly abundant and variable, with 27 clinical and four environmental pandemic serotypes identified in nine coastal provinces and two inland provinces (Beijing and Sichuan) (Figure 2). The most widely disseminated serotype of clinical isolates was O3:K6 (in 11 provinces), followed by O4:K68 (in eight provinces), O1:KUT (in five provinces), O1:K25 (in four provinces), and O1:K36 (in four provinces). The four environmental serotypes were identified in Shanghai (O1:KUT, O3:K6), Jiangsu (O3:K6, O4:K48), Zhejiang (O3:K6), and Guangdong (O4:K9). In addition, the sources of the environmental O3:K6 isolates were diverse in Jiangsu province (Table 2).

**Table 2:** **Sero-prevalence of pandemic *V. parahaemolyticus* isolates from Chinese samples**.

**Serotypes (*n*#)**	**Region (*n*) (year of isolation)**
**CLINICAL (27 SEROTYPES)**	
O1:K25 (*n* = 14)	Guangdong (*n* = 6) (2006–2012), Jiangsu (*n* = 3) (2007, 2014), Shanghai (*n* = 3) (2006–2007, 2010–2012), Taiwan (*n* = 2) (1998)
O1:K26 (*n* = 1)	Jiangsu (*n* = 1) (2007)
	Guangdong (*n* = 2) (2007–2012), Jiangsu (*n* = 1) (2007), Shanghai (*n* = 2) (2009–2012), Zhejiang (*n* = 22) (2009–2012)
O1:K5 (*n* = 2)	Jiangsu (*n* = 1) (2007), Zhejiang (*n* = 1) (2009)
O1:K56 (*n* = 3)	Jiangsu (*n* = 2) (2008), Shanghai (*n* = 1) (2010)
O1:KUT (*n* = 18)	Guangdong (*n* = 2) (2007–2012), Jiangsu (*n* = 4) (2005–2008), Liaoning (*n* = 1) (2010), Shanghai (*n* = 4) (2006–2007, 2009–2012), Zhejiang (*n* = 7) (2003, 2010, 2012)
O1:K6 (*n* = 2)	Shanghai (*n* = 2) (2007)
O1:K68 (*n* = 2)	Guangdong (*n* = 1) (2006–2011), Shanghai (*n* = 1) (2007)
O10:K60 (*n* = 1)	Shanghai (*n* = 1) (2010–2012)
O11:K36 (*n* = 6)	Guangdong (*n* = 6) (2006–2011)
O2:K68 (*n* = 1)	Shanghai (*n* = 1) (2007)
O3:K25 (*n* = 3)	Jiangsu (*n* = 2) (2007), Liaoning (*n* = 1) (2010)
O3:K29 (*n* = 1)	Jiangsu (*n* = 1) (2007)
O3:K3 (*n* = 1)	Shanghai (*n* = 1) (2010–2012)
O3:K6 (*n* = 103)	Beijing (*n* = 2) (2010), Guangdong (*n* = 9) (2006–2012), Guangxi (*n* = 4) (2003–2005, 2007), Hebei (*n* = 1) (2007), Jiangsu (*n* = 9) (2006–2009, 2014), Liaoning (*n* = 5) (2005, 2010), Shandong (*n* = 1) (2007), Shanghai (*n* = 12) (2006–2007, 2009–2012), Sichuan (*n* = 2) (2009), Zhejiang (*n* = 54) (2002–2003, 2006, 2009–2012), Taiwan (*n* = 4) (1996, 1998–1999, 2006)
O3:K68 (*n* = 5)	Jiangsu (*n* = 2) (2006), Shanghai (*n* = 1) (2006), Zhejiang (*n* = 2) (2010)
O3:K8 (*n* = 2)	Shanghai (*n* = 1) (2009–2011), Zhejiang (*n* = 1) (2010)
O3:KUT (*n* = 9)	Guangdong (*n* = 1) (2007–2012), Jiangsu (*n* = 1) (2009), Shanghai (*n* = 7) (2009–2012)
O4:K1 (*n* = 1)	Zhejiang (*n* = 1) (2010)
O4:K48 (*n* = 2)	Jiangsu (*n* = 1) (2005–2008), Zhejiang (*n* = 1) (2010)
O4:K68 (*n* = 68)	Guangdong (*n* = 7) (2007–2012), Jiangsu (*n* = 1) (2008), Liaoning (*n* = 1) (2010), Shanghai (*n* = 11) (2006–2007, 2010–2012), Zhejiang (*n* = 12) (2010–2012), Taiwan (*n* = 1) (1999)
O4:K8 (*n* = 23)	Guangdong (*n* = 1) (2007–2011), Shanghai (*n* = 1) (2006), Zhejiang (*n* = 21) (2006, 2009–2010, 2012)
O4:KUT (*n* = 4)	Shanghai (*n* = 1) (2007), Zhejiang (*n* = 3) (2006, 2010)
O5:K68 (*n* = 2)	Guangdong (*n* = 2) (2007–2012)
O5:KUT (*n* = 1)	Shanghai (*n* = 1) (2010–2012)
OUT:K22 (*n* = 1)	Zhejiang (*n* = 1) (2010)
OUT:KUT (*n* = 8)	Zhejiang (*n* = 8) (2010, 2012)
**ENVIRONMENTAL (4 SEROTYPES)**	
O1:KUT (*n* = 1)	Shanghai (*n* = 1) (2006-Ribbon fish)
O3:K6 (*n* = 11)	Shanghai (*n* = 1) (2011, Environmental isolates), Jiangsu (*n* = 9) (2005–2008, foodborne isolates-Bombay duck, Clam. Crab, Metapenaeus ensis, Qingchuan fish, Ribbon fish, Salmon, Seajelly, Thamnaconus septentrionalis), Zhejiang (*n* = 1) (2007, Ribbon fish)
O4:K48 (*n* = 1)	Jiangsu (*n* = 1) (2005–2008, foodborne isolates-Metapenaeus ensis)
O4:K9 (*n* = 1)	Guangdong (*n* = 1) (2006, Food isolates)

*n#, number of collected isolates*.

When sorting the isolates by the year of isolation, we found that only the O3:K6, O1:K25, O4:K68, and O1:KUT4 serotypes had been reported prior to 2005. However, from 2006 to 2012, as many as 24 more pandemic serotypes were detected (Table 3). This may be a significant period for the spreading of pandemic serotypes in China, but underreporting and possible study deviation should also be considered. As these factors directly affect the accuracy of our conjecture. O3:K6, first discovered in 1996, was detected each year from 2002 to 2012.

**Table 3:**
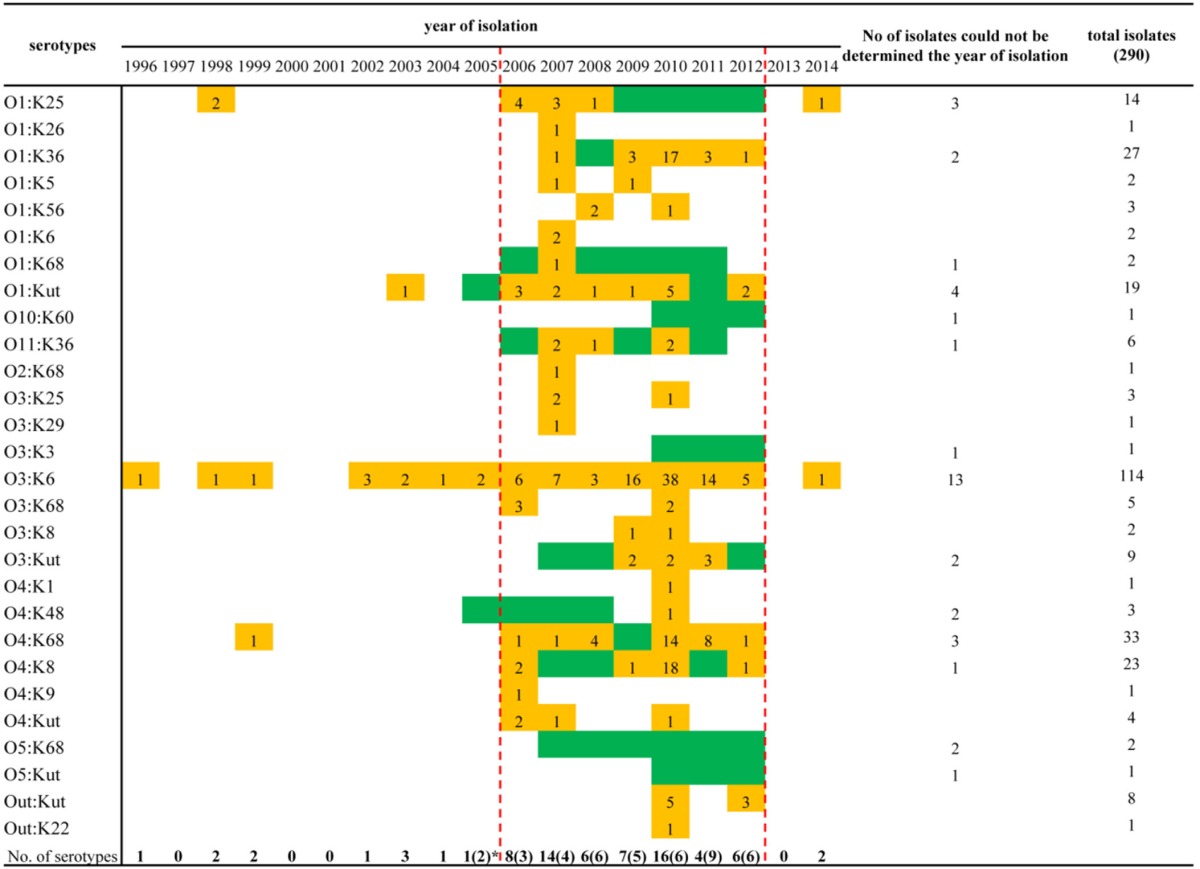
**Chronology of appearance of pandemic *V. parahaemolyticus* serotypes**.

Yellow marker indicates the year in which pandemic serotype was detected, the number in the yellow marker represent number of isolates; The green marker indicates that it's uncertain whether or not a pandemic serotype was detected in the corresponding year, in the original literature, the author only gave a time range; ^*^The number in the brackets means the serotypes that we cannot know whether or not they were identified in the corresponding years.

### Genetic differentiation of the pandemic isolates

Among the 290 collected pandemic isolates, 28 isolates was not typed by MLST analysis in their original studies, the other 262 isolates were involved the MLST analysis in the current study (Figure 1). These pandemic isolates exhibited 15 STs, revealing high genetic diversity (Table 4). The sequence variation of the isolates in Guangdong (11 STs) was significantly higher than that in other provinces (Figure 1). ST3 was the only sequence type that caused a wide range of infections in as many as ten provinces. Only three sequence types (ST3, ST192, and ST305) had ever been identified in environmental isolates.

**Table 4:** **Sequence types, allele profiles, and serotypes of pandemic *V. paraheamolyticus* isolates**.

**MLST assay**								**Serotypes**	
**ST (*n*^#^)**	***dnaE***	***dtdS***	***gyrB***	***pntA***	***pyrC***	***recA***	***tnaA***	**Clinical (*n*)**	**Environmental (*n*)**
ST3 (221)	3	4	4	29	4	19	22	O1:K25 (10), O1:K36 (24), O1:K56 (2), O1:K6 (2), O1:K68 (2), O1:Kut (15), O11:K36 (5), O2:K68 (1), O3:K25 (3), O3:K6 (86), O3:K68 (5), O3:Kut (7), O3:K8 (1), O4:K1 (1), O4:K48 (2), O4:K68 (29), O4:K8 (3), O5:K68 (2), O4:K48, Out: Kut (8), Out: K22 (1)	O3:K6 (11), O4:K48 (1)
ST192 (3)	3	4	126	29	4	19	22	O1:K26 (1), O1:Kut (1)	O4:K9 (1)
ST227 (1)	3	4	4	29	22	19	22	O3:K6 (1)	–
ST305 (3)	3	147	4	93	4	19	22	O1:K25 (2)	O1:Kut (1)
ST431 (2)	3	4	225	29	4	19	22	O3:K6 (2)	–
ST435 (2)	3	4	4	29	4	31	22	O3:K6 (2)	–
ST487 (1)	3	4	48	29	4	19	22	O3:K6 (1)	–
ST489 (1)	3	4	4	29	197	19	22	O3:K6 (1)	–
ST492 (1)	3	4	4	29	4	189	22	O1:K36 (1)	–
ST496 (1)	3	4	4	29	4	19	142	O11:K36 (1)	–
ST526 (1)	3	4	108	29	4	19	22	O3:K6 (1)	–
ST672 (1)	1	4	147	29	4	19	22	O3:K6 (1)	–
ST787 (2)	3	4	4	29	48	19	22	O4:K68 (2)	–
ST302 (1)	27	106	127	152	54	124	101	O4:Kut (1)	–
ST88 (21)	11	48	48	26	48	43	26	O4:K8 (21)	−

*n^#^, the number of isolates*.

*Compared with ST3, the changed allele types in other STs were marked by shadow. The number of alleles in each gene ranged from eight (gyrB) to three (dnaE, dtdS, pntA, and tnaA)*.

**Figure 3:**
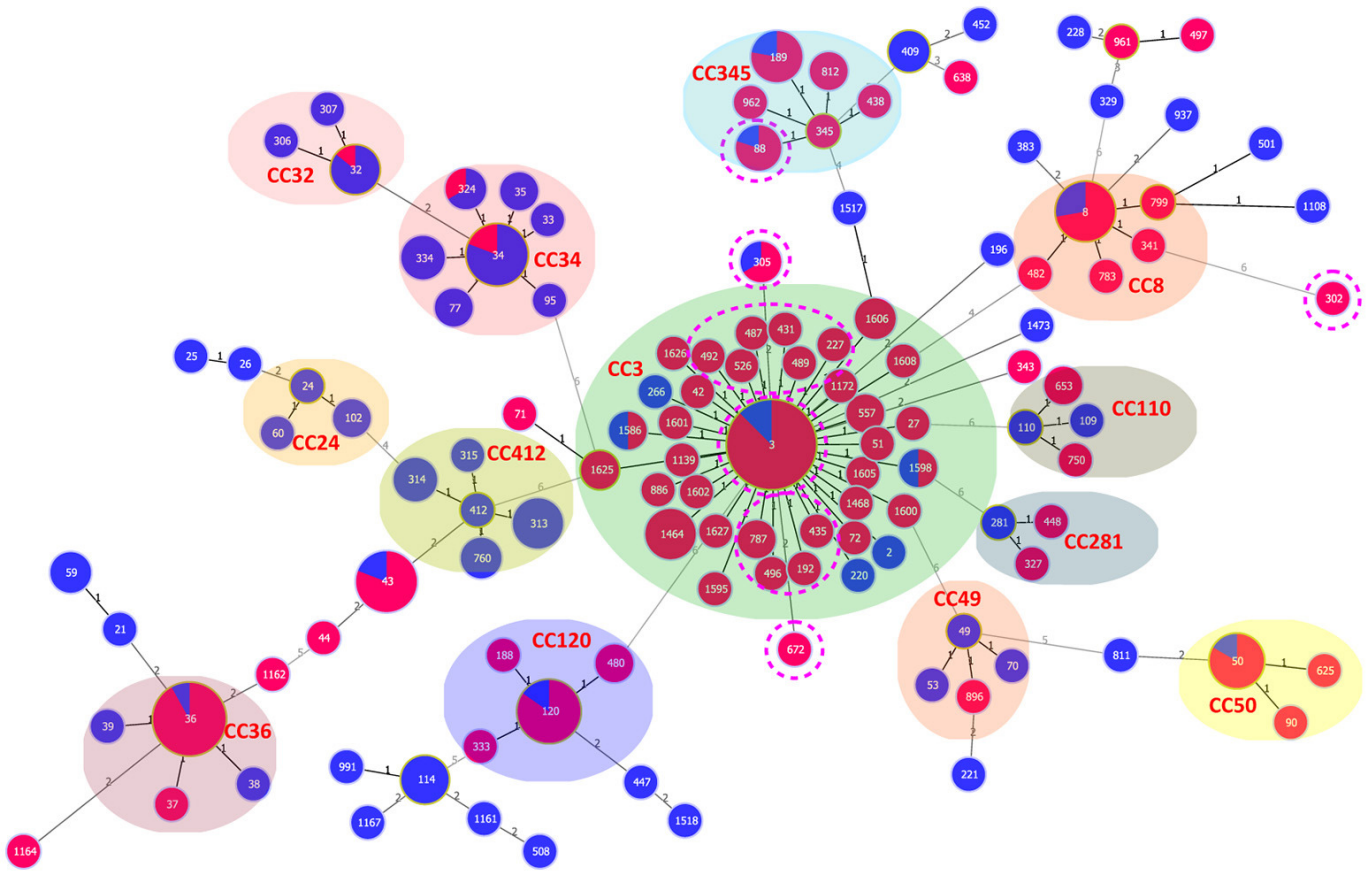
**goeBURST full MST of the STs shows the clonal diversity of Chinese clinical and environmental pandemic *V. parahaemolyticus* isolates: clinical (red) and environmental (blue)**. The pandemic STs in China are denoted by pink dotted circles. Other STs were selected from the public MLST database (https://pubmlst.org/vparahaemolyticus/) to help us analyze the cluster relationship of pandemic STs in this study. The number of different alleles is presented between STs connected via a line. The circle size varies according to the frequency of STs. Each shaded area represents a unique clone complex.

Ten of the 15 pandemic STs could be classified in CC3 (Figure 3). ST305 and ST672 were DLVs of ST3. Pandemic ST88 was found only in Zhejiang province during 2010–2012 and it belongs to CC345, not CC3. ST302 originated from in the Shanghai province and was identified as a singleton with no relationship to other STs.

### The association between pandemic STs and serotypes

The pandemic isolates within ST3 comprised 21 clinical and two environmental serotypes (O3:K6 and O4:K48), thus exhibiting high serotypic diversity (Table 4). ST192 included isolates that belong to O1:K26 (clinical), and O1: KUT (clinical) and O4:K9 (environmental) serotypes. ST305 consisted of two serotypes, O1:K25 (clinical) and O4:Kut (environmental). The remaining STs consisted of a single serotype. From another perspective, the pandemic O3:K6 serotype was shared by eight different STs (ST3, ST227, ST431, ST435, ST487, ST489, ST526, and ST672). O1:Kut isolates were divided into three STs (ST3, ST192, and ST305). Other serotypes were clustered in no more than two different pandemic STs.

### Antibiotic susceptibility profile of different pandemic isolates

**Figure 4:**
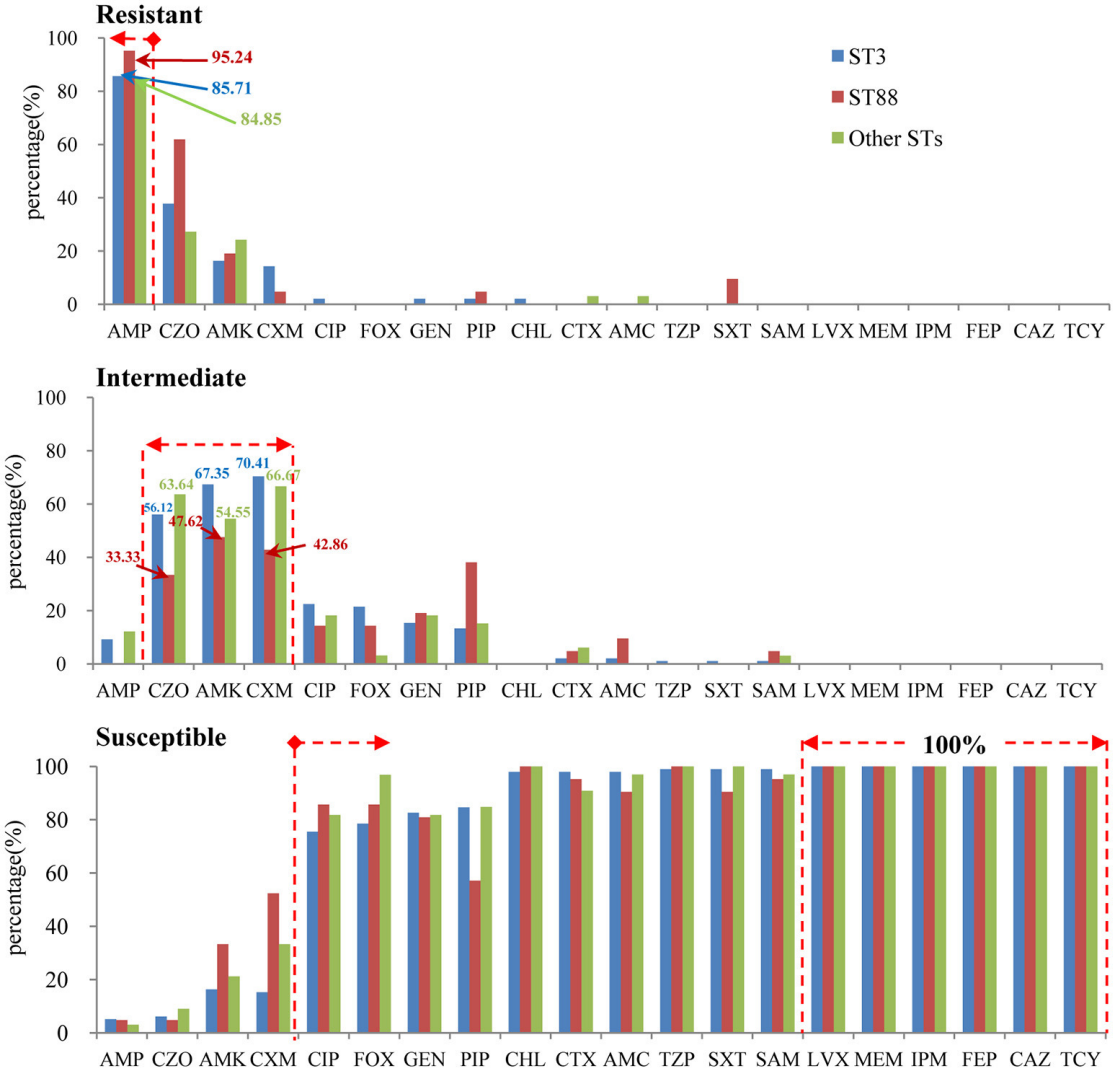
**The antimicrobial profiles within different STs (pandemic: ST3 and ST88, non-pandemic: other STs)**.

The results of antimicrobial susceptibilities of the 98 pandemic ST3 isolates, 21 pandemic ST88 isolates and 33 non-pandemic isolates are shown in Table S2. The results indicate similar antimicrobial profiles between isolates within different STs. In other words, there were not obvious differences in their resistance spectrums (Figure 4). Specifically, the isolates were mostly resistant to ampicillin (AMP) (95.2% of ST88, 85.7% of ST3, and 84.9% of other STs), and showed intermediate resistance to cefazolin (CZO), Amikacin (AMK), and Cefuroxime (CXM). It is reassuring that the isolates were susceptible to the majority of antibiotic tested, and all of them were susceptible to levofloxacin (LVX), meropenem (MEM), imipenem (IPM), cefepime (FEP), ceftazidime (CAZ), and tetracyclines (TCY). Additionally, there was no multidrug resistant bacteria (MDR) found.

## Discussion

As an emerging public health concern, pandemic *V. parahaemolyticus* infection has attracted wide attention from scientists (Jun et al., 2014; Velazquez-Roman et al., 2014; de Jesús Hernández-Díaz et al., 2015). The present study has provided an overview of the prevalence of pandemic isolates of *V. parahaemolyticus* in both clinical and environmental samples collected from multiple Chinese studies. We demonstrated that these pandemic isolates showed high serotypic and genetic diversity. The O3:K6 pandemic isolates (persistent for 11 years from 2002 to 2012) spread across 11 provinces indicate that the pandemic clone has been endemically established in China. Continued monitoring of antibiotic resistance patterns in pandemic isolates is urgently needed to avoid the excessive misuse of antibiotics, although most of the isolates tested in this study only showed high resistance to ampicillin.

The serovariants of this pandemic clone were abundant and variable. From 1996 to 2007, up to 22 pandemic serovariants were identified around the world (Nair et al., 2007), suggesting that the pandemic isolates could easily adapt new serotypes to survive in new environments. In this study, we found that 27 pandemic clinical serotypes and four environmental serotypes have been identified in the collected Chinese isolates. This is a significant number when compared to the number of serotypes found in other countries or regions (Nair et al., 2007; Pazhani et al., 2014; Velazquez-Roman et al., 2014), although the regional persistence of O3:K6 pandemic isolates has also been discovered in many other countries, such as Peru (Gil et al., 2007), Chile (Ansede-Bermejo et al., 2010), Japan (Okuda et al., 1997), India (Pazhani et al., 2014), and Thailand (Mala et al., 2016). To the best of our knowledge, no study has explained why serotypes of the pandemic clone are so diverse in China. Researchers around the world have made some progress in finding the cause of such serodiversity of the pandemic clone. Such research has determined that the O- and K-antigens are mostly mutated concurrently by horizontal gene transfer (HGT) (Okura et al., 2008), and it is speculated that new serovariants have emerged from the pandemic O3:K6 strains via replacement of the putative O and K antigen gene clusters (Okura et al., 2008; Harth et al., 2009; Chen et al., 2010). This is important for the survival of the pandemic clone in the face of changing external environments and host immunological resistance.

The analyzed isolates in this study were distributed in regions where the differences of temperature and other environmental factors are vast. Therefore, we suspect that the strains must adapt to different living environments in the transmission process by altering their biological properties more frequently. Making serological changes may be one of the most efficient ways for this to be accomplished. However, the specific mechanism of serotype conversion is not yet known. The highest priority is currently to continuously monitor the emergence of new serovariants to prevent the pandemic strains from causing outbreaks along the coastline and spreading to other countries and regions.

MLST is known to provide greater resolution than stereotyping. In a previous study, we provided an extensive review of isolates found in Chinese patients, and the results indicated a high degree of genetic diversity and a complicated population structure of *V. parahaemolyticus* (Han et al., 2015). With the present MLST study, we intended to demonstrate genetic similarities or differences among the pandemic isolates identified from different sources. As expected, we found that most of the pandemic STs were classified into the same clonal complex (CC3). However, some molecular divergence was also found. All the pandemic ST88 in Zhejiang province were assigned to CC345 (not CC3), and ST302, which originated in Shanghai province, was identified as a singleton with no relationship to other pandemic STs. Singletons do not seem to belong to the same clone as other pandemic isolates, but they do share the pandemic traits (toxRS/*new*+, *tdh*+, and *trh*−).

Chen et al. demonstrated the isolates of pandemic ST302 were clustered with other pandemic isolates based on other molecular typing methods, such as enterobacterial repetitive intergenic consensus sequence PCR (ERIC-PCR) (Chen et al., 2012). Similarly, in our pulsed field gel electrophoresis (PFGE) analysis (data not shown), the pandemic isolates of ST88 and ST3 shared the same PFGE profile. Thus, we observed that current typing and clustering methods may lead to controversial results, making it difficult to draw conclusions.

Therefore, a combined application of several molecular typing techniques should be considered in epidemiological investigations of *V. parahaemolyticus* pandemic isolates. As explained before (in Materials and Methods section), WGS is the best way to accurately portray the evolution and population structure of *V. parahaemolyticus* isolates at the molecular level (Cui et al., 2015; Haendiges et al., 2015), but the high cost limits its popularity in the analysis of large quantities of specimens.

Another important aspect of this study was the investigation of the antimicrobial susceptibility of different pandemic isolates. Our results revealed similar antibiotic susceptibility profiles in pandemic ST3, ST88, and non-pandemic isolates. This finding was similar to that in the work of Elmahdi et al. (2016). They concluded that the sampling location or month in which the samples were collected did not significantly impact *V. parahaemolyticus* resistance patterns because isolates from both environmental and clinical sources shared similar antibiotic resistance profiles. Unsurprisingly, the majority of the isolates tested in this study showed ampicillin resistance, which is very common in *V. parahaemolyticus* isolates recovered from different sources (Sun et al., 2013; de Jesús Hernández-Díaz et al., 2015; Elmahdi et al., 2016; Mala et al., 2016). This result suggests that ampicillin should have a negligible role in the treatment of *V. parahaemolyticus* infection. In fact, a survey conducted in the United States showed that very high *V. parahaemolyticus* ampicillin resistance could be traced as far back as 1978 (Blake et al., 1979).

In contrast, most of the isolates tested were sensitive to the majority of antibiotics tested, and all isolates were susceptible to LVX, MEM, IPM, FEP, and CAZ. This result suggests that these drugs can be used as an alternative antibiotic therapy. It must be noted that recently isolated *V. parahaemolyticus* strains, including pandemic strains, have displayed resistance to multiple antibiotics (Jun et al., 2012, 2014), which increases concerns about possible antibiotic treatment failure. Although we did not discover any multidrug resistant bacteria (MDR) isolates in this study, continued monitoring of pandemic strain susceptibility to antibiotic resistance is urgently needed to avoid the excessive misuse of antibiotics used to treat infections that pose threats to public health.

Our findings represent a comprehensive review of the pandemic *V. parahaemolyticus* O3:K6 and its serovariants by thoroughly assessing an extensive collection of clinical and environmental pandemic isolates from multiple Chinese studies. High levels of serotypic and genetic diversity in the pandemic clone are found, which suggests that the involved regions are becoming important reservoirs for the emergence of novel pandemic strains, which makes the clinical management of the infection and its prevention potentially challenging. Thus, we underscore the need for routine clinical and environmental monitoring to prevent pandemic *V. parahaemolyticus* infection and dissemination, including monitoring of antimicrobial response even though most current antimicrobial agents in routine use are effective. The mechanism in which the isolates undergo seroconversion with pandemic genetic marks warrants extended investigation.

## Author contributions

Conceived and designed the experiments: DH and CH. Performed the experiments: DH, FY, and HT. Analyzed the data: DH, CW, and PZ. Contributed reagents/materials/analysis tools: DH and CR. Wrote the paper: DH.

### Conflict of interest statement

The authors declare that the research was conducted in the absence of any commercial or financial relationships that could be construed as a potential conflict of interest.
