# Supplementary material for: Spreading of Pandemic Vibrio parahaemolyticus O3:K6 and Its Serovariants: A Re-analysis of Strains Isolated from Multiple Studies
Source: Front Cell Infect Microbiol. 2017 May 18;7:188. doi: 10.3389/fcimb.2017.00188 (PMC5435814; doi:10.3389/fcimb.2017.00188)
Supplement: Supplementary file 1 [file Table1.DOCX]

Table S1. The representative clinical and environmental *V. parahaemolyticus* isolates with pandemic genetic marks (*toxRS*/new+, *tdh*+ and *trh*-) collected in China used in this study

| **Id** | **Isolate** | **Region** | **Year** | | **Serotype** | **Source** | **STs** | | **Reference** |
| --- | --- | --- | --- | --- | --- | --- | --- | --- | --- |
| 1 | V285 | Jiangsu | 2007 | | O1:K25 | clinical | ST3 | | (Chao et al., 2011) |
| 2 | V488 | Jiangsu | 2008 | | O1:K56 | clinical | ST3 | | (Chao et al., 2011) |
| 3 | V510 | Jiangsu | 2009 | | O3:Kut | clinical | ST3 | | (Chao et al., 2011) |
| 4 | V402 | Jiangsu | 2008 | | O1:Kut | clinical | ST192 | | (Chao et al., 2011) |
| 5 | V251 | Jiangsu | 2006 | | O3:K6 | clinical | ST3 | | (Chao et al., 2011) |
| 6 | V264 | Jiangsu | 2006 | | O3:K6 | clinical | ST3 | | (Chao et al., 2011) |
| 7 | V267 | Jiangsu | 2006 | | O1:Kut | clinical | ST3 | | (Chao et al., 2011) |
| 8 | V275 | Jiangsu | 2007 | | O1:K36 | clinical | ST3 | | (Chao et al., 2011) |
| 9 | V276 | Jiangsu | 2007 | | O3:K6 | clinical | ST3 | | (Chao et al., 2011) |
| 10 | V283 | Jiangsu | 2007 | | O3:K25 | clinical | ST3 | | (Chao et al., 2011) |
| 11 | V263 | Jiangsu | 2006 | | O3:K68 | clinical | ST3 | | (Chao et al., 2011) |
| 12 | V258 | Jiangsu | 2006 | | O3:K68 | clinical | ST3 | | (Chao et al., 2011) |
| 13 | V327 | Jiangsu | 2007 | | O1:K26 | clinical | ST192 | | (Chao et al., 2011) |
| 14 | V336 | Jiangsu | 2007 | | O3:K6 | clinical | ST3 | | (Chao et al., 2011) |
| 15 | V350 | Jiangsu | 2008 | | O3:K6 | clinical | ST3 | | (Chao et al., 2011) |
| 16 | V504 | Jiangsu | 2009 | | O3:K6 | clinical | ST3 | | (Chao et al., 2011) |
| 17 | V506 | Jiangsu | 2009 | | O3:K6 | clinical | ST227 | | (Chao et al., 2011) |
| 18 | V252 | Jiangsu | 2008 | | O4:K68 | clinical | ST3 | | (Chao et al., 2011) |
| 19 | V323 | Jiangsu | 2006 | | O1:Kut | clinical | ST3 | | (Chao et al., 2009) |
| 20 | V377 | Jiangsu | 2008 | | O1:K56 | clinical | ST3 | | (Chao et al., 2009) |
| 21 | V283 | Jiangsu | 2007 | | O3:K25 | clinical | ST3 | | (Chao et al., 2009) |
| 22 | unassigned | Jiangsu | 2005-2008 | | O4:K48 | clinical | ST3 | | (Chao et al., 2009) |
| 23 | 1 | Guangdong | 2006 | | O1:K25 | clinical | ST3 | | (Ju et al., 2015) |
| 24 | W1 | Guangdong | 2006 | | O1:K25 | clinical | ST3 | | (Ju et al., 2015) |
| 25 | 34 | Guangdong | 2006 | | O1:K25 | clinical | ST3 | | (Ju et al., 2015) |
| 26 | HY20 | Guangdong | 2007 | | O11:K36 | clinical | ST3 | | (Ju et al., 2015) |
| 27 | W50 | Guangdong | 2007 | | O11:K36 | clinical | ST3 | | (Ju et al., 2015) |
| 28 | 3' | Guangdong | 2008 | | O11:K36 | clinical | ST3 | | (Ju et al., 2015) |
| 29 | 12月1日 | Guangdong | 2010 | | O11:K36 | clinical | ST3 | | (Ju et al., 2015) |
| 30 | 3 | Guangdong | 2006 | | O3:K6 | clinical | ST3 | | (Ju et al., 2015) |
| 31 | V-173 | Guangdong | 2010 | | O3:K6 | clinical | ST3 | | (Ju et al., 2015) |
| 32 | 10' | Guangdong | 2008 | | O4:K68 | clinical | ST3 | | (Ju et al., 2015) |
| 33 | GM7 | Guangdong | 2008 | | O4:K68 | clinical | ST3 | | (Ju et al., 2015) |
| 34 | GM6 | Guangdong | 2008 | | O4:K68 | clinical | ST3 | | (Ju et al., 2015) |
| 35 | 82 | Beijing | 2010 | | O3:K6 | clinical | ST431 | | (Fan et al., 2013) |
| 36 | 32502 | Liaoning | 2010 | | O3:K25 | clinical | ST3 | | (Li, 2012) |
| 37 | 32505 | Liaoning | 2010 | | O3:K6 | clinical | ST3 | | (Li, 2012) |
| 38 | 32506 | Liaoning | 2010 | | O4:K68 | clinical | ST3 | | (Li, 2012) |
| 39 | 32510 | Liaoning | 2010 | | O3:K6 | clinical | ST3 | | (Li, 2012) |
| 40 | 32512 | Liaoning | 2010 | | O1:Kut | clinical | ST3 | | (Li, 2012) |
| 41 | 32513 | Liaoning | 2010 | | O3:K6 | clinical | ST3 | | (Li, 2012) |
| 42 | lg1390 | Liaoning | 2010 | | O3:K6 | clinical | ST3 | | (Li, 2012) |
| 43 | rfh015 | Beijing | 2010 | | O3:K6 | clinical | ST3 | | (Li, 2012) |
| 44 | SH11VP046 | Shanghai | 2011 | | O3:Kut | clinical | ST3 | | (Li, 2012) |
| 45 | SH11VP048 | Shanghai | 2011 | | O3:K6 | clinical | ST435 | | (Li, 2012) |
| 46 | SH11VP053 | Shanghai | 2011 | | O4:K68 | clinical | ST3 | | (Li, 2012) |
| 47 | SH11VP071 | Shanghai | 2011 | | O3:K6 | environmental | ST3 | | (Li, 2012) |
| 48 | SH11VP076 | Shanghai | 2011 | | O3:K6 | clinical | ST3 | | (Li, 2012) |
| 49 | SH11VP084 | Shanghai | 2011 | | O4:K68 | clinical | ST3 | | (Li, 2012) |
| 50 | SH11VP085 | Shanghai | 2011 | | O4:K68 | clinical | ST3 | | (Li, 2012) |
| 51 | SH11VP086 | Shanghai | 2011 | | O3:K6 | clinical | ST3 | | (Li, 2012) |
| 52 | SH11VP087 | Shanghai | 2011 | | O3:Kut | clinical | ST3 | | (Li, 2012) |
| 53 | SH11VP088 | Shanghai | 2011 | | O3:Kut | clinical | ST3 | | (Li, 2012) |
| 54 | SH11VP090 | Shanghai | 2011 | | O4:K68 | clinical | ST3 | | (Li, 2012) |
| 55 | SH11VP091 | Shanghai | 2011 | | O4:K68 | clinical | ST3 | | (Li, 2012) |
| 56 | SH11VP094 | Shanghai | 2011 | | O4:K68 | clinical | ST3 | | (Li, 2012) |
| 57 | SH11VP095 | Shanghai | 2011 | | O4:K68 | clinical | ST3 | | (Li, 2012) |
| 58 | SH11VP097 | Shanghai | 2011 | | O3:K6 | clinical | ST3 | | (Li, 2012) |
| 59 | SH11VP158 | Shanghai | 2010 | | O3:Kut | clinical | ST3 | | (Li, 2012) |
| 60 | SH11VP166 | Shanghai | 2010 | | O3:Kut | clinical | ST3 | | (Li, 2012) |
| 61 | SH11VP168 | Shanghai | 2010 | | O3:K6 | clinical | ST3 | | (Li, 2012) |
| 62 | SH11VP257 | Shanghai | 2011 | | O3:K6 | clinical | ST3 | | (Li, 2012) |
| 63 | Unassigned | Guangdong | 2007-2011 | | O3:K6 | clinical | ST431 | | (Xiao, 2015) |
| 64 | Unassigned | Guangdong | 2007-2011 | | O3:K6 | clinical | ST3 | | (Xiao, 2015) |
| 65 | Unassigned | Guangdong | 2007-2011 | | O1:Kut | clinical | ST3 | | (Xiao, 2015) |
| 66 | Unassigned | Guangdong | 2007-2011 | | O4:K68 | clinical | ST3 | | (Xiao, 2015) |
| 67 | Unassigned | Guangdong | 2007-2011 | | O3:Kut | clinical | ST3 | | (Xiao, 2015) |
| 68 | Unassigned | Guangdong | 2007-2011 | | O4:K8 | clinical | ST3 | | (Xiao, 2015) |
| 69 | Unassigned | Guangdong | 2007-2011 | | O5:K68 | clinical | ST3 | | (Xiao, 2015) |
| 70 | F10-105 | Guangdong | 2010 | | O4:K68 | clinical | ST787 | | (Xiao, 2015) |
| 71 | F10-106 | Guangdong | 2010 | | O4:K68 | clinical | ST787 | | (Xiao, 2015) |
| 72 | Unassigned | Guangdong | 2010 | | O3:K6 | clinical | ST435 | | (Xiao, 2015) |
| 73 | F6 | Shanghai | 2006 | | O1:Kut | Ribbon fish | ST305 | | (Chen et al., 2012) |
| 74 | F13 | Zhejiang | 2007 | | O3:K6 | Ribbon fish | ST3 | | (Chen et al., 2012) |
| 75 | P6 | Shanghai | 2006 | | O1:K25 | clinical | ST305 | | (Chen et al., 2012) |
| 76 | P7 | Shanghai | 2006 | | O3:K6 | clinical | ST3 | | (Chen et al., 2012) |
| 77 | P10 | Shanghai | 2006 | | O3:K68 | clinical | ST3 | | (Chen et al., 2012) |
| 78 | P32 | Shanghai | 2006 | | O4:K8 | clinical | ST3 | | (Chen et al., 2012) |
| 79 | P35 | Shanghai | 2006 | | O3:K6 | clinical | ST3 | | (Chen et al., 2012) |
| 80 | P39 | Shanghai | 2006 | | O4:K68 | clinical | ST3 | | (Chen et al., 2012) |
| 81 | P43 | Shanghai | 2007 | | O4:Kut | clinical | ST302 | | (Chen et al., 2012) |
| 82 | P50 | Shanghai | 2007 | | O3:K6 | clinical | ST3 | | (Chen et al., 2012) |
| 83 | P83 | Shanghai | 2007 | | O1:K6 | clinical | ST3 | | (Chen et al., 2012) |
| 84 | P85 | Shanghai | 2007 | | O1:K68 | clinical | ST3 | | (Chen et al., 2012) |
| 85 | P86 | Shanghai | 2007 | | O1:Kut | clinical | ST3 | | (Chen et al., 2012) |
| 86 | P90 | Shanghai | 2007 | | O1:K6 | clinical | ST3 | | (Chen et al., 2012) |
| 87 | P167 | Shanghai | 2007 | | O1:Kut | clinical | ST3 | | (Chen et al., 2012) |
| 88 | QD2 | Shandong | 2007 | | O3:K6 | clinical | ST3 | | (Chen et al., 2012) |
| 89 | P196 | Shanghai | 2007 | | O4:K68 | clinical | ST3 | | (Chen et al., 2012) |
| 90 | P446 | Shanghai | 2007 | | O2:K68 | clinical | ST3 | | (Chen et al., 2012) |
| 91 | P447 | Shanghai | 2007 | | O1:K25 | clinical | ST3 | | (Chen et al., 2012) |
| 92 | NB755 | Zhejiang | 2006 | | O4:Kut | clinical | Untyped | | (Chen et al., 2012) |
| 93 | NB761 | Zhejiang | 2006 | | O4:Kut | clinical | Untyped | | (Chen et al., 2012) |
| 94 | WX1461 | Jiangsu | 2014 | | O1:K25 | clinical | ST3 | | (Li et al., 2016) |
| 95 | WX1478 | Jiangsu | 2014 | | O3:K6 | clinical | ST3 | | (Li et al., 2016) |
| 96 | Unassigned | Guangdong | 2007-2012 | | O3:K6 | clinical | ST3 | | (Li et al., 2014) |
| 97 | Unassigned | Guangdong | 2007-2012 | | O1:Kut | clinical | ST3 | | (Li et al., 2014) |
| 98 | Unassigned | Guangdong | 2007-2012 | | O1:K36 | clinical | ST3 | | (Li et al., 2014) |
| 99 | Unassigned | Guangdong | 2007-2012 | | O4:K68 | clinical | ST3 | | (Li et al., 2014) |
| 100 | Unassigned | Guangdong | 2007-2012 | | O5:K68 | clinical | ST3 | | (Li et al., 2014) |
| 101 | Unassigned | Guangdong | 2007-2012 | | O1:K25 | clinical | ST3 | | (Li et al., 2014) |
| 102 | Unassigned | Guangdong | 2006–2011 | | O1:K68 | clinical | ST3 | | (Sun et al., 2015) |
| 103 | Unassigned | Guangdong | 2006–2011 | | O11:K36 | clinical | ST3 | | (Sun et al., 2015) |
| 104 | Unassigned | Guangdong | 2007-2012 | | O1:K25 | clinical | ST3 | | (Li et al., 2014) |
| 105 | VP11153 | Guangdong | 2008 | | O3:K6 | clinical | ST487 | | (Li et al., 2014) |
| 106 | VP11155 | Guangdong | 2008 | | O3:K6 | clinical | ST489 | | (Li et al., 2014) |
| 107 | VP11113 | Guangdong | 2011 | | O3:K6 | clinical | ST526 | | (Li et al., 2014) |
| 108 | Unassigned | Guangdong | 2006 | | O4:K9 | Food | ST192 | | (Sun et al., 2015) |
| 109 | Unassigned | Guangdong | 2008 | | O1:K25 | Clinical | ST305 | | (Sun et al., 2015) |
| 110 | Unassigned | Guangdong | 2010 | | O1:K36 | Clinical | ST492 | | (Sun et al., 2015) |
| 111 | Unassigned | Guangdong | 2010 | | O11:K36 | Clinical | ST496 | | (Sun et al., 2015) |
| 112 | S063 | Taiwan | 1998 | | O1:K25 | Clinical | ST3 | | (Han et al., 2008) |
| 113 | S064 | Taiwan | 1998 | | O3:K6 | Clinical | ST3 | | (Han et al., 2008) |
| 114 | S065 | Taiwan | 1998 | | O1:K25 | Clinical | ST3 | | (Han et al., 2008) |
| 115 | S078 | Taiwan | 1999 | | O3:K6 | Clinical | ST3 | | (Han et al., 2008) |
| 116 | S090 | Taiwan | 1999 | | O4:K68 | Clinical | ST3 | | (Han et al., 2008) |
| 117 | S092 | Taiwan | 1996 | | O3:K6 | Clinical | ST3 | | (Han et al., 2008) |
| 118 | S126 | Taiwan | 2006 | | O3:K6 | Clinical | ST3 | | (Han et al., 2008) |
| 119 | S133 | Liaoning | 2005 | | O3:K6 | Clinical | ST3 | | (Han et al., 2008) |
| 120 | S135 | Guangxi | 2003 | | O3:K6 | Clinical | ST3 | | (Han et al., 2008) |
| 121 | S136 | Guangxi | 2004 | | O3:K6 | Clinical | ST3 | | (Han et al., 2008) |
| 122 | S137 | Guangxi | 2005 | | O3:K6 | Clinical | ST3 | | (Han et al., 2008) |
| 123 | S138 | Hebei | 2007 | | O3:K6 | Clinical | ST3 | | (Han et al., 2008) |
| 124 | Unassigned | Shanghai | 2010-2012 | | O3:K6 | Clinical | Untyped | | (Chen et al., 2014) |
| 125 | Unassigned | Shanghai | 2010-2012 | | O4:K68 | Clinical | Untyped | | (Chen et al., 2014) |
| 126 | Unassigned | Shanghai | 2010-2012 | | O1:K25 | Clinical | Untyped | | (Chen et al., 2014) |
| 127 | Unassigned | Shanghai | 2010-2012 | | O1:K36 | Clinical | Untyped | | (Chen et al., 2014) |
| 128 | Unassigned | Shanghai | 2010-2012 | | O1:Kut | Clinical | Untyped | | (Chen et al., 2014) |
| 129 | Unassigned | Shanghai | 2010-2012 | | O3:Kut | Clinical | Untyped | | (Chen et al., 2014) |
| 130 | Unassigned | Shanghai | 2010-2012 | | O5:Kut | Clinical | Untyped | | (Chen et al., 2014) |
| 131 | Unassigned | Shanghai | 2010-2012 | | O3:K3 | Clinical | Untyped | | (Chen et al., 2014) |
| 132 | Unassigned | Shanghai | 2010-2012 | | O10:K60 | Clinical | Untyped | | (Chen et al., 2014) |
| 133 | Z10-8 | Zhejiang | 2010 | | O4:K48 | Clinical | ST3 | | (Chen et al., 2016) |
| 134 | Z10-16 | Zhejiang | 2010 | | O1:K36 | Clinical | ST3 | | (Chen et al., 2016) |
| 135 | Z10-17 | Zhejiang | 2010 | | O4:K68 | Clinical | ST3 | | (Chen et al., 2016) |
| 136 | Z10-22 | Zhejiang | 2010 | | O3:K6 | Clinical | ST3 | | (Chen et al., 2016) |
| 137 | Z10-27 | Zhejiang | 2010 | | O1:Kut | Clinical | ST3 | | (Chen et al., 2016) |
| 138 | Z10-32 | Zhejiang | 2010 | | O4:K68 | Clinical | ST3 | | (Chen et al., 2016) |
| 139 | Z10-39 | Zhejiang | 2010 | | O3:K6 | Clinical | ST3 | | (Chen et al., 2016) |
| 140 | Z10-40 | Zhejiang | 2010 | | O4:K1 | Clinical | ST3 | | (Chen et al., 2016) |
| 141 | Z10-41 | Zhejiang | 2010 | | O3:K6 | Clinical | ST3 | | (Chen et al., 2016) |
| 142 | Z10-50 | Zhejiang | 2010 | | O3:K6 | Clinical | ST3 | | (Chen et al., 2016) |
| 143 | Z10-51 | Zhejiang | 2010 | | O1:K36 | Clinical | ST3 | | (Chen et al., 2016) |
| 144 | Z10-54 | Zhejiang | 2010 | | O4:K68 | Clinical | ST3 | | (Chen et al., 2016) |
| 145 | Z09-58 | Zhejiang | 2009 | | O3:K6 | Clinical | ST3 | | (Chen et al., 2016) |
| 146 | Z09-68 | Zhejiang | 2009 | | O3:K6 | Clinical | ST3 | | (Chen et al., 2016) |
| 147 | Z09-72 | Zhejiang | 2009 | | O3:K6 | Clinical | ST3 | | (Chen et al., 2016) |
| 148 | Z09-74 | Zhejiang | 2009 | | O3:K6 | Clinical | ST3 | | (Chen et al., 2016) |
| 149 | Z09-75 | Zhejiang | 2009 | | O3:K6 | Clinical | ST3 | | (Chen et al., 2016) |
| 150 | Z09-79 | Zhejiang | 2009 | | O3:K6 | Clinical | ST3 | | (Chen et al., 2016) |
| 151 | Z10-91 | Zhejiang | 2010 | | O4:K68 | Clinical | ST3 | | (Chen et al., 2016) |
| 152 | Z10-97 | Zhejiang | 2010 | | O4:K68 | Clinical | ST3 | | (Chen et al., 2016) |
| 153 | Z10-101 | Zhejiang | 2010 | | O3:K6 | Clinical | ST3 | | (Chen et al., 2016) |
| 154 | Z10-116 | Zhejiang | 2010 | | O4:K68 | Clinical | ST3 | | (Chen et al., 2016) |
| 155 | Z10-117 | Zhejiang | 2010 | | O3:K6 | Clinical | ST3 | | (Chen et al., 2016) |
| 156 | Z10-119 | Zhejiang | 2010 | | O4:K68 | Clinical | ST3 | | (Chen et al., 2016) |
| 157 | Z10-123 | Zhejiang | 2010 | | O3:K6 | Clinical | ST3 | | (Chen et al., 2016) |
| 158 | Z10-125 | Zhejiang | 2010 | | O1:Kut | Clinical | ST3 | | (Chen et al., 2016) |
| 159 | Z10-132 | Zhejiang | 2010 | | O1:K36 | Clinical | ST3 | | (Chen et al., 2016) |
| 160 | Z10-148 | Zhejiang | 2010 | | O3:K68 | Clinical | ST3 | | (Chen et al., 2016) |
| 161 | Z10-157 | Zhejiang | 2010 | | O4:K68 | Clinical | ST3 | | (Chen et al., 2016) |
| 162 | Z10-160 | Zhejiang | 2010 | | O3:K6 | Clinical | ST3 | | (Chen et al., 2016) |
| 163 | Z10-168 | Zhejiang | 2010 | | O4:K68 | Clinical | ST3 | | (Chen et al., 2016) |
| 164 | Z10-173 | Zhejiang | 2010 | | O3:K68 | Clinical | ST3 | | (Chen et al., 2016) |
| 165 | Z10-186 | Zhejiang | 2010 | | O1:K36 | Clinical | ST3 | | (Chen et al., 2016) |
| 166 | Z10-228 | Zhejiang | 2010 | | Out: Kut | Clinical | ST3 | | (Chen et al., 2016) |
| 167 | Z10-273 | Zhejiang | 2010 | | Out:K22 | Clinical | ST3 | | (Chen et al., 2016) |
| 168 | Z10-279 | Zhejiang | 2010 | | O1:K36 | Clinical | ST3 | | (Chen et al., 2016) |
| 169 | Z10-289 | Zhejiang | 2010 | | O4:K68 | Clinical | ST3 | | (Chen et al., 2016) |
| 170 | Z10-346 | Zhejiang | 2010 | | O1:Kut | Clinical | ST3 | | (Chen et al., 2016) |
| 171 | Z10-350 | Zhejiang | 2010 | | O1:Kut | Clinical | ST3 | | (Chen et al., 2016) |
| 172 | Z10-352 | Zhejiang | 2010 | | O3:K6 | Clinical | ST3 | | (Chen et al., 2016) |
| 173 | Z10-353 | Zhejiang | 2010 | | O1:K36 | Clinical | ST3 | | (Chen et al., 2016) |
| 174 | Z10-381 | Zhejiang | 2010 | | O1:K36 | Clinical | ST3 | | (Chen et al., 2016) |
| 175 | Z10-415 | Zhejiang | 2010 | | O3:K6 | Clinical | ST3 | | (Chen et al., 2016) |
| 176 | Z10-416 | Zhejiang | 2010 | | O3:K6 | Clinical | ST3 | | (Chen et al., 2016) |
| 177 | Z10-417 | Zhejiang | 2010 | | O3:K6 | Clinical | ST3 | | (Chen et al., 2016) |
| 178 | Z10-418 | Zhejiang | 2010 | | O3:K6 | Clinical | ST3 | | (Chen et al., 2016) |
| 179 | Z10-420 | Zhejiang | 2010 | | O3:K6 | Clinical | ST3 | | (Chen et al., 2016) |
| 180 | Z10-448 | Zhejiang | 2010 | | O3:K8 | Clinical | ST3 | | (Chen et al., 2016) |
| 181 | Z10-484 | Zhejiang | 2010 | | O4:K68 | Clinical | ST3 | | (Chen et al., 2016) |
| 182 | Z10-550 | Zhejiang | 2010 | | O4:K8 | Clinical | ST88 | | (Chen et al., 2016) |
| 183 | Z10-591 | Zhejiang | 2010 | | O1:K36 | Clinical | ST3 | | (Chen et al., 2016) |
| 184 | Z10-593 | Zhejiang | 2010 | | O4:K8 | Clinical | ST88 | | (Chen et al., 2016) |
| 185 | Z10-594 | Zhejiang | 2010 | | O4:K8 | Clinical | ST88 | | (Chen et al., 2016) |
| 186 | Z10-598 | Zhejiang | 2010 | | O3:K6 | Clinical | ST3 | | (Chen et al., 2016) |
| 187 | Z10-599 | Zhejiang | 2010 | | O3:K6 | Clinical | ST672 | | (Chen et al., 2016) |
| 188 | Z10-601 | Zhejiang | 2010 | | O3:K6 | Clinical | ST3 | | (Chen et al., 2016) |
| 189 | Z10-604 | Zhejiang | 2010 | | O3:K6 | Clinical | ST3 | | (Chen et al., 2016) |
| 190 | Z10-609 | Zhejiang | 2010 | | Out: Kut | Clinical | ST3 | | (Chen et al., 2016) |
| 191 | Z10-626 | Zhejiang | 2010 | | O4:K8 | Clinical | ST88 | | (Chen et al., 2016) |
| 192 | Z10-631 | Zhejiang | 2010 | | O4:K8 | Clinical | ST88 | | (Chen et al., 2016) |
| 193 | Z10-646 | Zhejiang | 2010 | | O4:K8 | Clinical | ST88 | | (Chen et al., 2016) |
| 194 | Z10-647 | Zhejiang | 2010 | | O4:K8 | Clinical | ST88 | | (Chen et al., 2016) |
| 195 | Z10-651 | Zhejiang | 2010 | | O4:K8 | Clinical | ST88 | | (Chen et al., 2016) |
| 196 | Z10-652 | Zhejiang | 2010 | | O4:K8 | Clinical | ST88 | | (Chen et al., 2016) |
| 197 | Z10-662 | Zhejiang | 2010 | | O4:K8 | Clinical | ST88 | | (Chen et al., 2016) |
| 198 | Z10-702 | Zhejiang | 2010 | | O3:K6 | Clinical | ST3 | | (Chen et al., 2016) |
| 199 | Z10-704 | Zhejiang | 2010 | | Out: Kut | Clinical | ST3 | | (Chen et al., 2016) |
| 200 | Z10-726 | Zhejiang | 2010 | | O1:K36 | Clinical | ST3 | | (Chen et al., 2016) |
| 201 | Z10-753 | Zhejiang | 2010 | | O3:K6 | Clinical | ST3 | | (Chen et al., 2016) |
| 202 | Z10-755 | Zhejiang | 2010 | | O3:K6 | Clinical | ST3 | | (Chen et al., 2016) |
| 203 | Z10-759 | Zhejiang | 2010 | | O3:K6 | Clinical | ST3 | | (Chen et al., 2016) |
| 204 | Z10-797 | Zhejiang | 2010 | | Out: Kut | Clinical | ST3 | | (Chen et al., 2016) |
| 205 | Z10-808 | Zhejiang | 2010 | | O4:K8 | Clinical | ST88 | | (Chen et al., 2016) |
| 206 | Z10-815 | Zhejiang | 2010 | | O1:K36 | Clinical | ST3 | | (Chen et al., 2016) |
| 207 | Z10-821 | Zhejiang | 2010 | | O4:K8 | Clinical | ST88 | | (Chen et al., 2016) |
| 208 | Z10-833 | Zhejiang | 2010 | | O1:K36 | Clinical | ST3 | | (Chen et al., 2016) |
| 209 | Z10-840 | Zhejiang | 2010 | | O4:K8 | Clinical | ST88 | | (Chen et al., 2016) |
| 210 | Z10-842 | Zhejiang | 2010 | | O4:K8 | Clinical | ST88 | | (Chen et al., 2016) |
| 211 | Z10-852 | Zhejiang | 2010 | | O4:K8 | Clinical | ST88 | | (Chen et al., 2016) |
| 212 | Z10-905 | Zhejiang | 2010 | | O3:K6 | Clinical | ST3 | | (Chen et al., 2016) |
| 213 | Z10-906 | Zhejiang | 2010 | | O3:K6 | Clinical | ST3 | | (Chen et al., 2016) |
| 214 | Z10-911 | Zhejiang | 2010 | | O4:K8 | Clinical | ST88 | | (Chen et al., 2016) |
| 215 | Z10-917 | Zhejiang | 2010 | | O1:K36 | Clinical | ST3 | | (Chen et al., 2016) |
| 216 | Z10-930 | Zhejiang | 2010 | | O3:K6 | Clinical | ST3 | | (Chen et al., 2016) |
| 217 | Z10-931 | Zhejiang | 2010 | | O1:K36 | Clinical | ST3 | | (Chen et al., 2016) |
| 218 | Z10-935 | Zhejiang | 2010 | | O4:K8 | Clinical | ST88 | | (Chen et al., 2016) |
| 219 | Z10-961 | Zhejiang | 2010 | | O3:K6 | Clinical | ST3 | | (Chen et al., 2016) |
| 220 | Z10-963 | Zhejiang | 2010 | | O1:K36 | Clinical | ST3 | | (Chen et al., 2016) |
| 221 | Z10-964 | Zhejiang | 2010 | | O3:K6 | Clinical | ST3 | | (Chen et al., 2016) |
| 222 | Z10-965 | Zhejiang | 2010 | | O1:K36 | Clinical | ST3 | | (Chen et al., 2016) |
| 223 | Z10-966 | Zhejiang | 2010 | | O3:K6 | Clinical | ST3 | | (Chen et al., 2016) |
| 224 | Z10-967 | Zhejiang | 2010 | | O4:Kut | Clinical | ST88 | | (Chen et al., 2016) |
| 225 | Z10-977 | Zhejiang | 2010 | | O1:K36 | Clinical | ST3 | | (Chen et al., 2016) |
| 226 | Z10-1193 | Zhejiang | 2009 | | O3:K6 | Clinical | ST3 | | (Chen et al., 2016) |
| 227 | Z09-1224 | Zhejiang | 2009 | | O4:K8 | Clinical | ST88 | | (Chen et al., 2016) |
| 228 | Z09-1226 | Zhejiang | 2009 | | O3:K6 | Clinical | ST3 | | (Chen et al., 2016) |
| 229 | Z09-1227 | Zhejiang | 2009 | | O3:K6 | Clinical | ST3 | | (Chen et al., 2016) |
| 230 | Z09-1228 | Zhejiang | 2009 | | O1:K36 | Clinical | ST3 | | (Chen et al., 2016) |
| 231 | Z10-1239 | Zhejiang | 2010 | | Out: Kut | Clinical | ST3 | | (Chen et al., 2016) |
| 232 | Z10-1242 | Zhejiang | 2010 | | O4:K8 | Clinical | ST88 | | (Chen et al., 2016) |
| 233 | Z11-151 | Zhejiang | 2011 | | O3:K6 | Clinical | ST3 | | (Chen et al., 2016) |
| 234 | **Z11-**232 | Zhejiang | 2011 | | O3:K6 | Clinical | ST3 | | (Chen et al., 2016) |
| 235 | Z11-239 | Zhejiang | 2011 | | O3:K6 | Clinical | ST3 | | (Chen et al., 2016) |
| 236 | Z11-255 | Zhejiang | 2011 | | O1:K36 | Clinical | ST3 | | (Chen et al., 2016) |
| 237 | Z11-256 | Zhejiang | 2011 | | O3:K6 | Clinical | ST3 | | (Chen et al., 2016) |
| 238 | Z11-258 | Zhejiang | 2011 | | O3:K6 | Clinical | ST3 | | (Chen et al., 2016) |
| 239 | Z11-261 | Zhejiang | 2011 | | O3:K6 | Clinical | ST3 | | (Chen et al., 2016) |
| 240 | Z11-381 | Zhejiang | 2011 | | O1:K36 | Clinical | ST3 | | (Chen et al., 2016) |
| 241 | Z11-416 | Zhejiang | 2011 | | O1:K36 | Clinical | ST3 | | (Chen et al., 2016) |
| 242 | Z11-582 | Zhejiang | 2011 | | O3:K6 | Clinical | ST3 | | (Chen et al., 2016) |
| 243 | Z12-117 | Zhejiang | 2012 | | O4:K8 | Clinical | ST88 | | (Chen et al., 2016) |
| 244 | Z12-131 | Zhejiang | 2012 | | O3:K6 | Clinical | ST3 | | (Chen et al., 2016) |
| 245 | Z12-143 | Zhejiang | 2012 | | O1:K36 | Clinical | ST3 | | (Chen et al., 2016) |
| 246 | Z12-169 | Zhejiang | 2012 | | O1:Kut | Clinical | ST3 | | (Chen et al., 2016) |
| 247 | Z12-181 | Zhejiang | 2012 | | O3:K6 | Clinical | ST3 | | (Chen et al., 2016) |
| 248 | Z12-189 | Zhejiang | 2012 | | O3:K6 | Clinical | ST3 | | (Chen et al., 2016) |
| 249 | Z12-193 | Zhejiang | 2012 | | O3:K6 | Clinical | ST3 | | (Chen et al., 2016) |
| 250 | Z12-196 | Zhejiang | 2012 | | O3:K6 | Clinical | ST3 | | (Chen et al., 2016) |
| 251 | Z12-208 | Zhejiang | 2012 | | Out: Kut | Clinical | ST3 | | (Chen et al., 2016) |
| 252 | Z12-210 | Zhejiang | 2012 | | Out: Kut | Clinical | ST3 | | (Chen et al., 2016) |
| 253 | Z12-216 | Zhejiang | 2012 | | Out: Kut | Clinical | ST3 | | (Chen et al., 2016) |
| 254 | Z12-255 | Zhejiang | 2012 | | O1:Kut | Clinical | ST3 | | (Chen et al., 2016) |
| 255 | Z12-257 | Zhejiang | 2012 | | O4:K68 | Clinical | ST3 | | (Chen et al., 2016) |
| 256 | SH2009021 | Shanghai | 2009 | | O3:K8 | clinical | Untyped | | (Zhang et al., 2013) |
| 257 | SH2009017 | Shanghai | 2009 | | O1:Kut | clinical | Untyped | | (Zhang et al., 2013) |
| 258 | SH2011110 | Shanghai | 2011 | | O4:K68 | clinical | Untyped | | (Zhang et al., 2013) |
| 259 | SH2010106 | Shanghai | 2010 | | O1:K56 | clinical | Untyped | | (Zhang et al., 2013) |
| 260 | SH2009059 | Shanghai | 2009 | | O3:K6 | clinical | Untyped | | (Zhang et al., 2013) |
| 261 | SH2010010 | Shanghai | 2010 | | O3:K6 | clinical | Untyped | | (Zhang et al., 2013) |
| 262 | SH2009005 | Shanghai | 2009 | | O3:KUT | clinical | Untyped | | (Zhang et al., 2013) |
| 263 | SH2009009 | Shanghai | 2009 | | O1:K36 | clinical | ST3 | | (Zhang et al., 2013) |
| 264 | unassigned | Jiangsu | 2005-2008 | | O4:K48 | foodborne isolates-Metapenaeus ensis | ST3 | | (Chao et al., 2009) |
| 265 | unassigned | Jiangsu | 2005-2008 | | O3:K6 | foodborne isolates-Qingchuan fish | ST3 | | (Chao et al., 2009) |
| 266 | unassigned | Jiangsu | 2005-2008 | | O3:K6 | foodborne isolates-Ribbon fish | ST3 | | (Chao et al., 2009) |
| 267 | unassigned | Jiangsu | 2005-2008 | | O3:K6 | foodborne isolates-Crab | ST3 | | (Chao et al., 2009) |
| 268 | unassigned | Jiangsu | 2005-2008 | | O3:K6 | foodborne isolates-Metapenaeus ensis | ST3 | | (Chao et al., 2009) |
| 269 | unassigned | Jiangsu | 2005-2008 | | O3:K6 | foodborne isolates-Salmon | ST3 | | (Chao et al., 2009) |
| 270 | unassigned | Jiangsu | 2005-2008 | | O3:K6 | Foodborne isolates-Thamnaconus septentrionalis | ST3 | | (Chao et al., 2009) |
| 271 | unassigned | Jiangsu | 2005-2008 | | O3:K6 | foodborne isolates-Bombay duck | ST3 | | (Chao et al., 2009) |
| 272 | unassigned | Jiangsu | 2005-2008 | | O3:K6 | foodborne isolates-Clam | ST3 | | (Chao et al., 2009) |
| 273 | unassigned | Jiangsu | 2005-2008 | | O3:K6 | foodborne isolates-Seajelly | ST3 | | (Chao et al., 2009) |
| 274 | unassigned | Jiangsu | 2005-2008 | | O1:Kut | clinical | ST3 | | (Chao et al., 2009) |
| 275 | 07VP518 | Jiangsu | 2007 | | O3:K29 | clinical | Untyped | | (Li et al., 2014) |
| 276 | 09VP157 | Zhejiang | 2009 | | O3:K6 | clinical | Untyped | | (Li et al., 2014) |
| 277 | 07VP521 | Jiangsu | 2007 | | O1:K25 | clinical | Untyped | | (Li et al., 2014) |
| 278 | 09VP45 | Sichuan | 2009 | | O3:K6 | clinical | Untyped | | (Li et al., 2014) |
| 279 | 07VP541 | Jiangsu | 2009 | | O3:K6 | clinical | Untyped | | (Li et al., 2014) |
| 280 | 07VP155 | Guangxi | 2007 | | O3:K6 | clinical | Untyped | | (Li et al., 2014) |
| 281 | 09VP170 | Zhejiang | 2009 | | O1:K36 | clinical | Untyped | | (Li et al., 2014) |
| 282 | 07VP531 | Jiangsu | 2007 | | O1:K5 | clinical | Untyped | | (Li et al., 2014) |
| 283 | 09VP167 | Zhejiang | 2009 | | O1:K5 | clinical | Untyped | | (Li et al., 2014) |
| 284 | 09VP45 | Sichuan | 2009 | | O3:K6 | clinical | Untyped | | (Li et al., 2014) |
| 285 | HM15 | Zhejiang | 2002 | | O3:K6 | clinical | ST3 | | (Yu et al., 2011) |
| 286 | HM18 | Zhejiang | 2002 | O4:K8 | | clinical | | ST3 | (Yu et al., 2011) |
| 287 | HM23 | Zhejiang | 2002 | O3:K6 | | clinical | | ST3 | (Yu et al., 2011) |
| 288 | HM12 | Zhejiang | 2006 | O3:K6 | | clinical | | ST3 | (Yu et al., 2011) |
| 289 | ZJ3 | Zhejiang | 2003 | O1:Kut | | clinical | | ST3 | (Yu et al., 2011) |
| 290 | ZJ17 | Zhejiang | 2003 | O3:K6 | | clinical | | ST3 | (Yu et al., 2011) |

**References:**

Chao, G., Jiao, X., Zhou, X., Yang, Z., Huang, J., and Pan, Z., et al. (2009). Serodiversity, Pandemic O3:K6 Clone, Molecular Typing, and Antibiotic Susceptibility of Foodborne and ClinicalVibrio parahaemolyticus Isolates in Jiangsu, China. *Foodborne Pathogens and Disease,* 6(8), 1021-1028. doi: 10.1089/fpd.2009.0295.

Chao, G., Wang, F., Zhou, X., Jiao, X., Huang, J., and Pan, Z., et al. (2011). Origin of Vibrio parahaemolyticus O3:K6 pandemic clone. *Int J Food Microbiol,* 145(2-3), 459-463. doi: 10.1016/j.ijfoodmicro.2011.01.022.

Chen, W., Xie, Y., Xu, J., Wang, Q., Gu, M., and Yang, J., et al. (2012). Molecular typing of Vibrio parahaemolyticus isolates from the middle-east coastline of China. *International Journal of Food Microbiology,* 153(3), 402-412. doi: 10.1016/j.ijfoodmicro.2011.12.001.

Chen, Y., Chen, X., Yu, F., Wu, M., Wang, R., and Zheng, S., et al. (2016). Serology, virulence, antimicrobial susceptibility and molecular characteristics of clinical Vibrio parahaemolyticus strains circulating in southeastern China from 2009 to 2013. *Clinical Microbiology and Infection,* 22(3), 258-259. doi: 10.1016/j.cmi.2015.11.003

Han, H., Wong, H. C., Kan, B., Guo, Z., Zeng, X., and Yin, S., et al. (2008). Genome plasticity of Vibrio parahaemolyticus: microevolution of the 'pandemic group'. *BMC Genomics,* 9, 570. doi: 10.1186/1471-2164-9-570.

Ju, C., Yu, M., Huang, R., Luo, J., and Duan, Y. (2015). Genetic characterization of Vibrio parahaemolyticus O3: K6 serovariant isolated in Shenzhen. *Zhonghua Yu Fang Yi Xue Za Zhi,* 49(1), 21-25.

Li, J., Xue, F., Yang, Z., Zhang, X., Zeng, D., and Chao, G., et al. (2016). Vibrio parahaemolyticus Strains of Pandemic Serotypes Identified from Clinical and Environmental Samples from Jiangsu, China. *Front Microbiol,* 7, 787. doi: 10.3389/fmicb.2016.00787.

Li, Y., Xie, X., Shi, X., Lin, Y., Qiu, Y., and Mou, J., et al. (2014). Vibrio parahaemolyticus, Southern Coastal Region of China, 2007–2012. *Emerging Infectious Diseases,* 20(4), 685-688. doi: 10.3201/eid2004.130744.

Sun, H., Li, Y., Shi, X., Lin, Y., Qiu, Y., and Zhang, J., et al. (2015). Association of CRISPR/Cas Evolution withVibrio parahaemolyticus Virulence Factors and Genotypes. *Foodborne Pathogens and Disease,* 12(1), 68-73. doi: 10.1089/fpd.2014.1792.

Yu, Y., Hu, W., Wu, B., Zhang, P., Chen, J., and Wang, S., et al. (2011). Vibrio parahaemolyticus isolates from southeastern Chinese coast are genetically diverse with circulation of clonal complex 3 strains since 2002. *Foodborne Pathog Dis,* 8(11), 1169-1176. doi: 10.1089/fpd.2011.0865

Zhang, H., Sun, S., Shi, W., Cui, L., and Gu, Q. (2013). Serotype, Virulence, and Genetic Traits of Foodborne and Clinical Vibrio parahaemolyticus Isolates in Shanghai, China. *Foodborne Pathogens and Disease,* 10(9), 796-804. doi: 10.1089/fpd.2012.1378.

Chen H., Sheng Y., Song Y., Tu L., Zhang X and Chen M. (2014). Serotypes and molecular characteristics of Vibrio parahaemolyticus pandemic strains in Shanghai. *CHINESE JOUＲNAL OF FOOD HYGIENE,* 26(01), 5-9.

Fan Y., Zhu M., Shang X., Wang M., Huang Y and Gu H., et al. (2013). Virulence characteristics and multilocus sequence type of Vibrio parahaemolyticus isolated from clini. *Chin J Lab Med,* 36(6), 548-552. doi: 10.3760/cma.j.issn.1009-9158.2013.06.016.

Li J. (2012). Molecular Typing and Genetic Variation Analysis of Vibrio Parahaemolyticus isolates From Different Sources and Different Regions. [master’s thesis]. [Beijing (IL)]: PLA Academy of Military Science.

Li W., Mei L., Tang Z., Yang X., Li X and Pei X., et al. (2014). Analysis of molecular features of clinical Vibrio parahaemolyticus strains in China. *Chin J Prey Med,* 48(1), 44-52. doi: 10.3760/cma.j.issn.0253-9624.2014.01.010

Xiao N. (2015) Etiologic characteristics of Vibrio parahaemolyticus strains isolated from patients in Guangdong, 2007-2013. [master’s thesis]. [Guangzhou (IL)]: Southern Medical University.
